# A Novel 6500 V SiC Trench MOSFET with Integrated Unipolar Diode for Improved Third Quadrant and Switching Characteristics

**DOI:** 10.3390/mi15010092

**Published:** 2023-12-31

**Authors:** Hao Wu, Xuan Li, Xiaochuan Deng, Yangyang Wu, Jiawei Ding, Wensong Peng, Bo Zhang

**Affiliations:** 1 State Key Laboratory of Electronic Thin Films and Integrated Devices, University of Electronic Science and Technology of China, Chengdu 610054, China; jerrywhtc@163.com (H.W.); xcdeng@uestc.edu.cn (X.D.); wuyangyang_uestc@163.com (Y.W.); djfdjw@163.com (J.D.); zhangbo@uestc.edu.cn (B.Z.); 2 Shenzhen Institute for Advanced Study, University of Electronic Science and Technology of China, Shenzhen 518110, China; pengwensong_uestc@163.com

**Keywords:** SiC trench MOSFET, bipolar degradation, integrated unipolar diode, low switching loss

## Abstract

A 6500 V SiC trench MOSFET with integrated unipolar diode (UD-MOS) is proposed to improve reverse conduction characteristics, suppress bipolar degradation, and reduce switching loss. An N type base region under the trench dummy gate provides a low barrier path to suppress hole injection during the reverse conduction operation. The reverse conduction voltage *V*_ON_ is reduced to 1.11 V, and the reverse recovery charge (*Q*_RR_) is reduced to 1.22 μC/cm^2^. The gate-to-drain capacitance (*C*_GD_) and gate-to-source capacitance (*C*_GS_) of the UD-MOS are also reduced to improve switching loss due to the thick oxide layer between the trench gate and dummy gate. The proposed device exhibits an excellent loss-related figure of merit (FOM). It provides a high-voltage SiC MOSFET prototype with potential performance advantages for voltage source converter-based high voltage direct current applications.

## 1. Introduction

The voltage source converter-based high-voltage direct current (VSC-HVDC) transmission with high flexibility and controllability is a key approach to the construction of a high-proportion clean energy grid. The high-voltage (6500 V and above) SiC MOSFET is a promising candidate for VSC-HVDC transmission to increase power efficiency and reduce the volume of the system [1,2,3]. In the VSC, the freewheeling diode of SiC MOSFET operates during the dead time period. However, the utilization of the integrated PN body diode causes potential issues. Firstly, the reverse recovery charge *Q*_RR_ and reverse conduction voltage *V*_ON_ considerably increase loss. Secondly, the basic plane dislocations (BPDs) generate stacking faults (SFs) in the drift region by absorbing the energy from charge recombination [4], which degrades the *V*_ON_ and leakage current of SiC MOSFET [5]. For the 6500 V SiC MOSFET with a thicker drift region, many more SFs could be generated based on same initial BPDs density, spelling more serious bipolar degradation issues [6].

To prevent SiC MOSFET bipolar degradation issues, a common solution is utilizing the externally antiparallel Schottky barrier diode (SBD) for freewheeling operation [7], but this increases the parasitic parameters and size of the MOSFETs-based power module [8]. However, for the monolithic integrated diode schemes, SBD-integrated MOSFETs have been reported [9,10,11] and the source-controlled channel-diode-embedded SiC MOSFETs have been demonstrated [12,13], whereas Schottky contact and the uneven gate oxide layer-related reliability issues have yet to be introduced [14,15,16,17]. Gate-controlled channel-diode-embedded SiC MOSFETs have also been fabricated [18,19,20], which allow the forward and reverse conduction currents to share the same MOS-channel path. However, this brings a huge challenge to achieving a better trade-off between low reverse conduction voltage and reasonable threshold voltage [18].

In this paper, a 6500 V SiC trench MOSFET with integrated unipolar diode (UD-MOS) is proposed to improve reverse conduction characteristics, suppress bipolar degradation issue, and reduce switching loss. Compared with the asymmetric trench MOSFET (C-MOS), the performance with its operation mechanism of UD-MOS is demonstrated by numerical simulations, involving doping-dependent mobility, high-field saturation mobility, Shockley–Read–Hall (SRH) recombination, Auger recombination, incomplete ionization of impurities, and impact ionization models.

## 2. Device Structure and Mechanism

**Figure 1:**
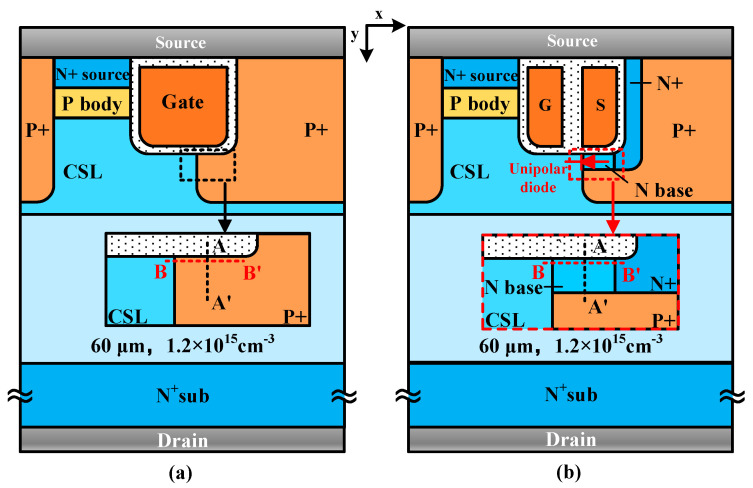
Schematic cross-section view of the 6500 V SiC (**a**) C-MOS and (**b**) UD-MOS.

The schematic cross-section view of the 6500 V SiC C-MOS and UD-MOS is shown in Figure 1. Compared with the C-MOS, the polysilicon gate of UD-MOS splits into two parts with a thick oxide layer. The left one is the true gate electrically connected to the gate electrode, while the right one is the dummy gate electrically connected to the source electrode. An N type region (i.e., N base region) beneath the dummy gate for electrons from the CSL region to N+ region, forms a unipolar diode (UD) as illustrated in Figure 1b. 

**Figure 2:**
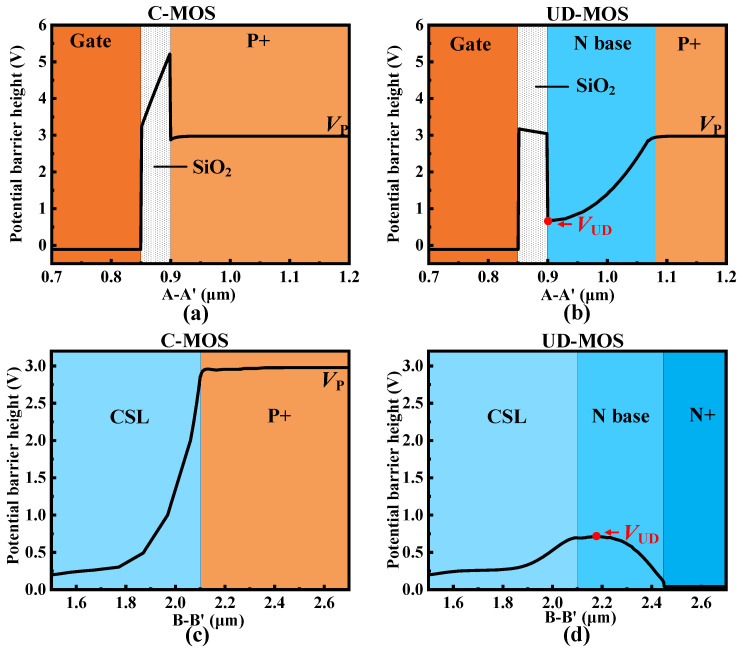
Potential barrier distribution at zero-bias (*V*_GS_ = 0 V and *V*_DS_ = 0 V) condition. (**a**) Along line A-A′ of the SiC C-MOS, (**b**) along line A-A′ of the SiC UD-MOS, (**c**) along line B-B′ of the SiC C-MOS, and (**d**) along line B-B′ of the SiC UD-MOS.

For the zero-bias condition, the potential barrier distribution of the integrated unipolar diode of UD-MOS is shown in Figure 2. Compared with the body diode of C-MOS, the decrease of potential barrier from the P+ region to the SiC/SiO_2_ interface (i.e., along line A′-A of Figure 1) makes a relatively low potential barrier (i.e., *V*_UD_) for electrons transported from the CSL to N+ region. It should be noted that the *V*_UD_ is still relatively higher than the potential barrier of the CSL region and the N+ region as shown in Figure 2d. In other words, the electrons cannot flow through the N base region to the N+ region under this condition.

**Figure 3:**
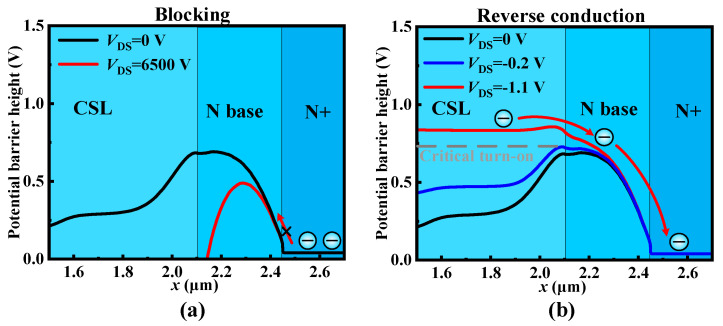
Potential barrier distribution along line B-B′ of the SiC UD-MOS. (**a**) The blocking condition and (**b**) the reverse conduction condition at *V*_GS_ = 0 V.

**Figure 4:**
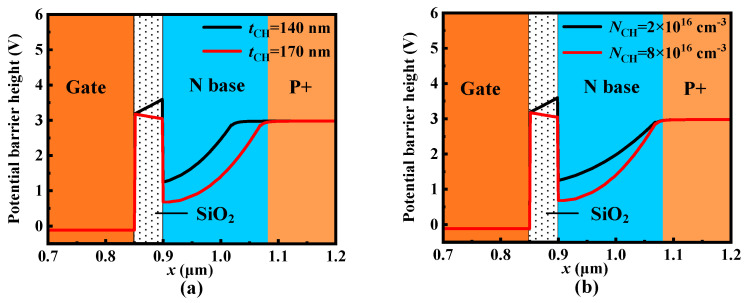
Effects of (**a**) *N*_CH_ and (**b**) *t*_CH_ on the potential barrier height along line A-A′ at zero-bias condition (*V*_GS_ = *V*_DS_ = 0 V).

When in blocking condition, although the increasing *V*_DS_ lowers the barrier of the CSL region and N base region due to the drain-induced barrier lowering effect, the *V*_UD_ is still high enough to ensure blocking capability, as shown in Figure 3a. When in the reverse conduction condition, the potential barrier of the CSL region is raised by the negative *V*_DS_. Once the potential barrier of the CSL region exceeds *V*_UD_, the electrons from the CSL region can flow through the N base region to the N+ region, as shown in Figure 3b. Therefore, the potential height of the N base region determines both the blocking and reverse conduction characteristics of UD-MOS. Furthermore, a barrier height analysis model is given here to inform the design of the *V*_UD_ as follows,

(1) $$V_{\mathrm{UD}}=V_{P}-\frac{\phi_{\mathrm{Si},\mathrm{SiC}}+V_{\mathrm{GS}}-\frac{qN_{\mathrm{CH}}t_{\mathrm{CH}}^{2}}{2\varepsilon_{\mathrm{SiC}}}}{\frac{\varepsilon_{\mathrm{SiC}}t_{\mathrm{ox}}}{\varepsilon_{\mathrm{ox}}t_{\mathrm{nch}}}+1}-\frac{qN_{\mathrm{CH}}t_{\mathrm{CH}}^{2}}{2\varepsilon_{\mathrm{SiC}}}$$

where *V*_P_ is the potential barrier height of P+ region, *ϕ*_Si,SiC_ is the work function difference between N-type polysilicon and the P+ region, *ε*_SiC_ is the dielectric constant of SiC, *ε*_ox_ is the dielectric constant of oxide, *q* is the elementary charge, *t*_CH_ is the thickness of the N base region, and *N*_CH_ is the doping concentration of the N base region, respectively. According to (1), even though the negative *V*_GS_ can enhance blocking capability by increasing the *V*_UD_, it also results in a high reverse conduction voltage *V*_ON_ of UD-MOS. Moreover, the positive *V*_GS_ even reduces the *V*_UD_ to make the unipolar diode turn on, causing the UD-MOS to lose gate control when in the forward conduction condition [17]. Therefore, the dummy gate not controlled by the gate electrode is introduced to guarantee both the forward and reverse conduction capability. Furthermore, the thickness *t*_CH_ and doping concentration *N*_CH_ of the N base region also affect the *V*_UD_. With the increase of *t*_CH_ and *N*_CH_, the *V*_UD_ decreases, as shown in Figure 4. The influence of breakdown voltage (*BV*) and *V*_ON_ on *t*_CH_ and *N*_CH_ is discussed further in the following section.

## 3. Results and Discussion

**Figure 5:**
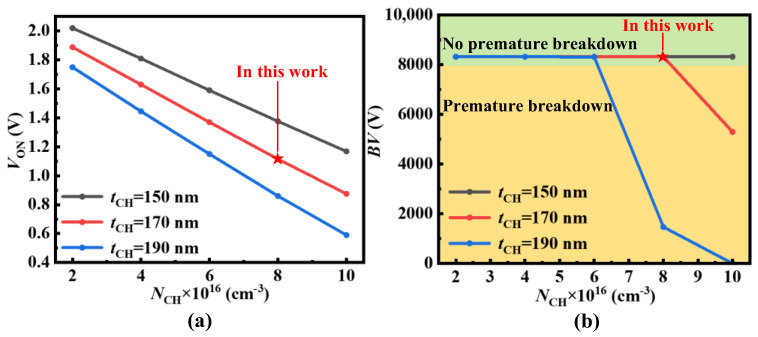
Effects of *t*_CH_ and *N*_CH_ on (**a**) *V*_ON_ and (**b**) *BV* of UD-MOS.

With the increase of *t*_CH_ and *N*_CH_, the *V*_ON_ (@*V*_GS_ = 0 V, *I*_DS_ = −3 A/cm^2^) and *BV* (@*I*_DS_ = 1 × 10^−8^ A/cm^2^) of UD-MOS decrease, as shown in Figure 5. It should be noted that the *V*_ON_ is mainly the voltage drop of the UD (i.e., *V*_UD_) and the thick epi-layer. The thicker *t*_CH_ and higher *N*_CH_ bring lower *V*_ON_, but also lead to premature breakdown. Therefore, considering both the *V*_ON_ and *BV* of the UD-MOS, the *t*_CH_ and *N*_CH_ are designed to be 170 nm and 8 × 10^16^ cm^−3^, respectively. The key structural parameters of the C-MOS and UD-MOS are shown in Table 1.

**Figure 6:**
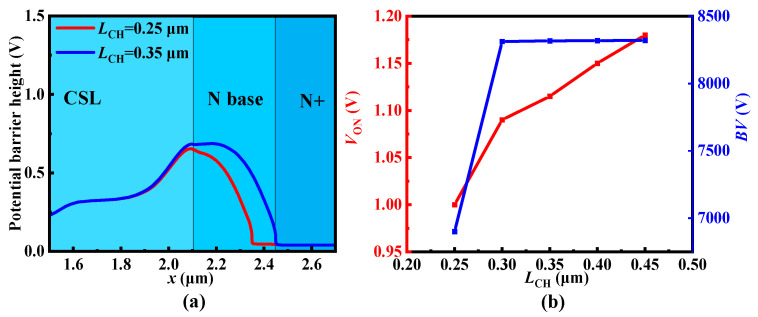
(**a**) Potential distribution in different *L*_CH_ and (**b**) effect of *L*_CH_ on *V*_ON_ and *BV*.

The effect of the N base length *L*_CH_ on *V*_UD_ is also discussed. With the narrowness of *L*_CH_, the potential of the N+ region influences the potential barrier of the N base as shown in Figure 6a. Although the lower *V*_UD_ helps to reduce the *V*_ON_, the *BV* is weakened at the same time, as shown in Figure 6b.

### 3.1. Static Characteristics

**Figure 7:**
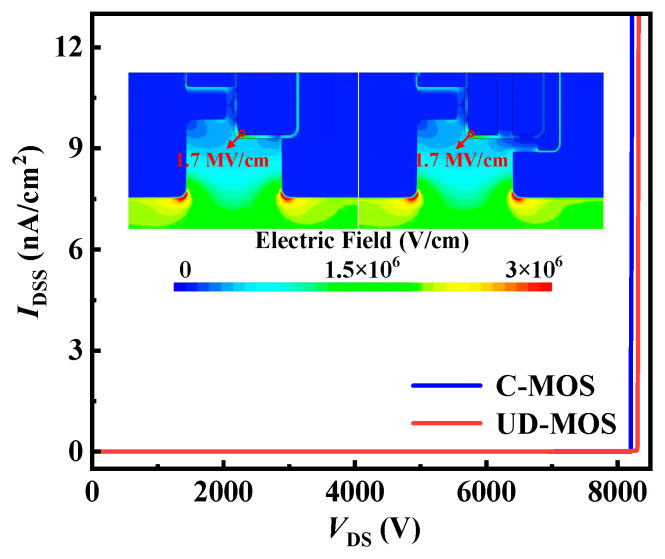
Blocking characteristics of the SiC C-MOS and UD-MOS. The insets show the electric field distributions in SiC MOSFET at *V*_DS_ = 6500 V.

Based on optimized *t*_CH_ and *N*_CH_, the SiC C-MOS and UD-MOS have a similar *BV,* as shown in Figure 7. The peak electric field in the gate oxide is less than 3 MV/cm, which ensures the long-term reliability of the gate oxide, as shown in the insets of Figure 7.

**Figure 8:**
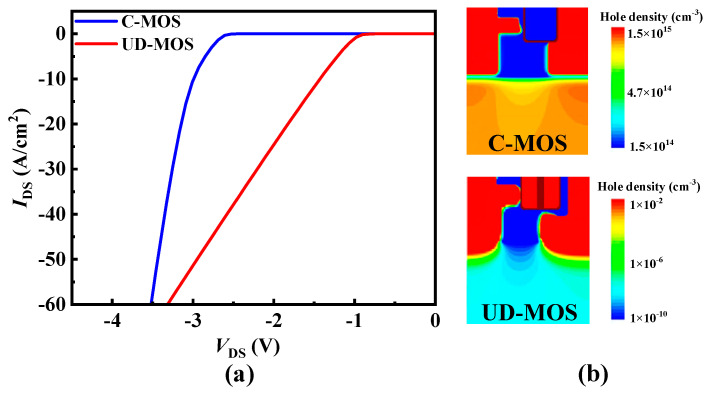
(**a**) Reverse conduction characteristics and (**b**) hole density distribution of the SiC C-MOS and UD-MOS at *V*_GS_ = 0 V and *I*_SD_ = 50 A/cm^2^.

Moreover, the *V*_ON_ of UD-MOS is −1.1 V, while the C-MOS is −2.8 V, as shown in Figure 8a. It should be noted that the integrated unipolar diode makes for lesser hole injection into the drift region when in the reverse conduction condition, as shown in Figure 8b, which effectively avoids the risk of bipolar degradation.

**Figure 9:**
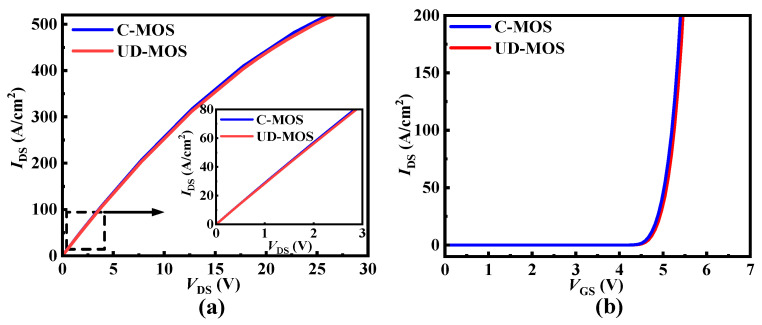
(**a**) Output characteristics at *V*_GS_ = 18 V and (**b**) transfer characteristics of the SiC C-MOS and UDMOS.

Even though the cell pitch of the UD-MOS is slightly larger than that of the C-MOS, the conduction capability of the UD-MOS is not degraded, because its channel density no longer dominates for high voltage SiC MOSFETs. The *R*_ON_ of UD-MOS and C-MOS are 35.48 mΩ·cm^2^ and 35.00 mΩ·cm^2^, respectively (@*I*_DS_ = 50 A/cm^2^), as shown in Figure 9a. The transfer characteristic of the UD-MOS is also not degraded, which shows nearly the same *V*_TH_ as the C-MOS, as shown in Figure 9b.

### 3.2. Dynamic Characteristics

**Figure 10:**
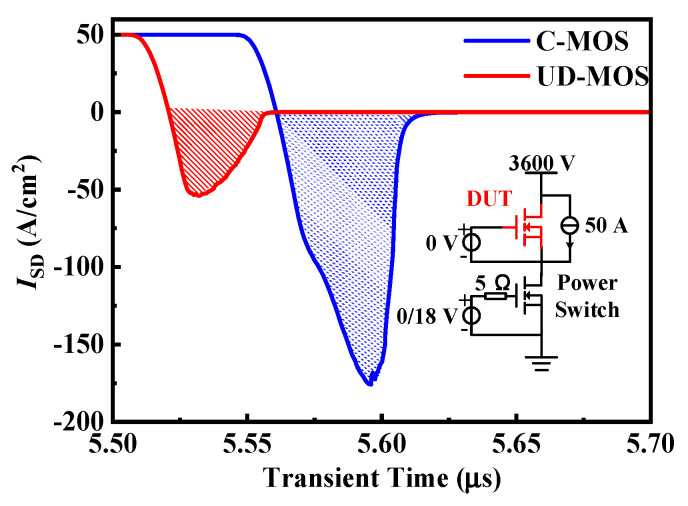
Reverse recovery characteristics of the body diode in the SiC C-MOS and UD-MOS.

The reverse recovery characteristics of the body diode in the SiC C-MOS and UD-MOS are compared, as shown in Figure 10. Thanks to no extraction of minority carrier during the reverse recovery process, the peak reverse recovery current (*I*_RRM_) and reverse recovery charge (*Q*_RR_) of UD-MOS are 54 A/cm^2^ and 1.04 μC/cm^2^, which are significantly reduced by 76% and 81%, respectively, compared to the C-MOS (*I*_RRM_ =176 A/cm^2^ and *Q*_RR_ =5 μC/cm^2^).

**Figure 11:**
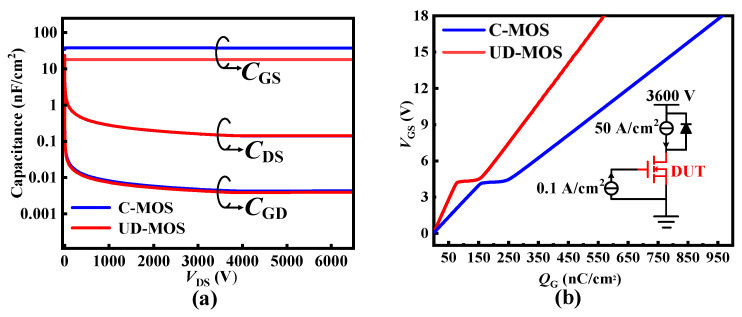
(**a**) Capacitance characteristics at *V*_GS_ = 0 V, *f* = 1 MHz and (**b**) *Q*_G_ for the SiC C-MOS and UD-MOS.

Moreover, the dummy gate of the UD-MOS reduces the effective overlapping area between gate and drain terminals, so that the gate-to-drain capacitance (*C*_GD_) is 4.01 pF/cm^2^ (@*V*_DS_ = 3600 V), which is reduced by 9.5% compared with the C-MOS. Meanwhile, due to the thick oxide layer between the trench gate and dummy gate as well as a slightly smaller overlapping area, the gate-to-source capacitance (*C*_GS_) of the UD-MOS is 18.1 nF/cm^2^, which is reduced by 52%. Accordingly, thanks to the smaller *C*_GD_ and *C*_GS_, the UD-MOS has a lower gate charge (*Q*_G_) of 566 nC/cm^2^ (@*V*_GS_ = 0 V–18 V) and gate-to-drain charge (*Q*_GD_) of 109 nC/cm^2^, as shown in Figure 11. Furthermore, considering conduction and dynamic capability, the UD-MOS exhibits a better loss-related figure of merit (FOM, i.e., *R*_ON_ *× Q*_GD_ [21]) of 3.87 mΩ·μC, which is 8.7% lower than that of the C-MOS.

**Figure 12:**
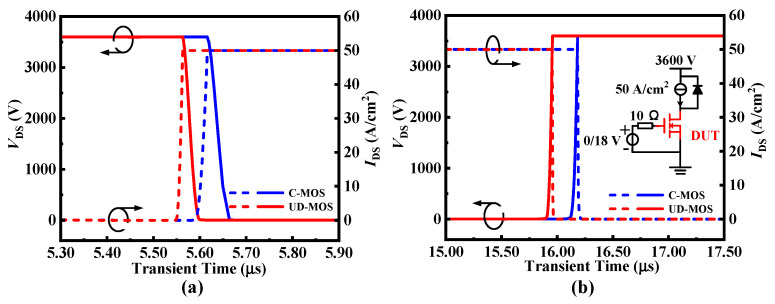
(**a**) Turn-on and (**b**) turn-off waveforms of the C-MOS and UD-MOS at *V*_DS_ = 3600 V and *I*_DS_ = 50 A/cm^2^.

The switching waveforms of the SiC C-MOS and DP-MOS are as shown in Figure 12. Benefitting from the reduced capacitances, the UD-MOS has lower turn-on loss (*E*_ON_) and turn-off loss (*E*_OFF_) of 3.80 mJ/cm^2^ and 3.36 mJ/cm^2^, which are 34% and 17% lower than that of the C-MOS, respectively.

**Figure 13:**
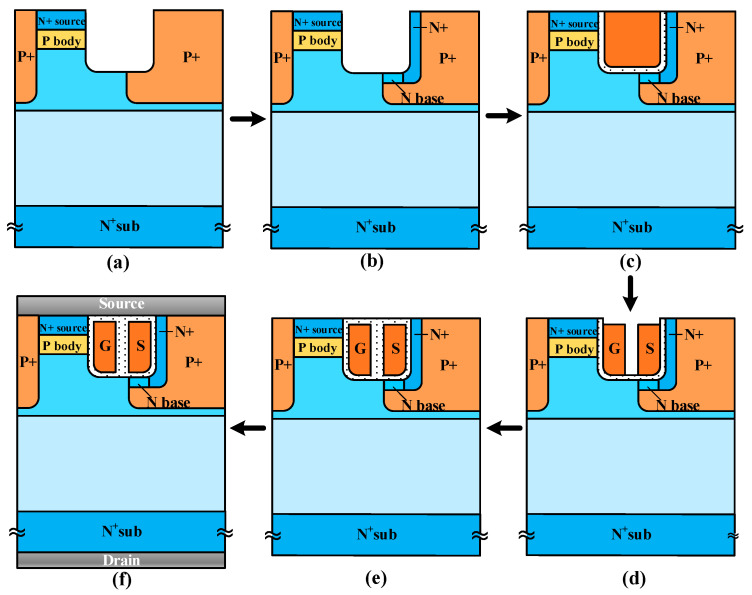
Brief fabrication process flow of the SiC UD-MOS. (**a**) Epitaxial growing, P body implantation, N+ source region implantation, trench etch and P+ region implantation, (**b**) N+ region and N base region implantation, (**c**) thermal oxidation and polysilicon gate deposition, (**d**) polysilicon etch by oxide sidewall spacer, (**e**) isolated oxidation deposition and (**f**) metallization.

One feasible process flow of the UD-MOS is presented, including (a) epitaxial growing, P body implantation, N+ source region implantation, trench etch and P+ region implantation, (b) N+ region and N base region implantation, (c) thermal oxidation and polysilicon gate deposition, (d) polysilicon etch, (e) isolated oxidation deposition and (f) metallization, as shown in Figure 13. Finally, Table 2 compares the main characteristics of the SiC UD-MOS and the C-MOS. The SiC UD-MOS exhibits superior performance due to the unipolar diode.

## 4. Conclusions

A novel 6500 V SiC trench UD-MOS is proposed with improved reverse conduction and switching characteristics. The grounded dummy gate causes the unipolar diode of the SiC UD-MOS to reduce *V*_ON_ to 1.1 V, which avoids the risk of bipolar degradation and reduces the parasitic capacitances with lower switching loss. The proposed UD-MOS provides a promising device prototype in VSC applications for HVDC transmission.

## Figures and Tables

**Table 1:** Key Structure Parameters of the SiC C-MOS and UD-MOS.

Parameters	UD-MOS	C-MOS
cell pitch (μm)	3.35	3.15
drift region thickness (μm)	60	60
drift region doping (cm^−3^)	1.2 × 10^15^	1.2 × 10^15^
polysilicon width (μm)	0.8	0.8
trench depth (μm)	0.9	0.9
trench width (μm)	1.1	0.9
oxide thickness (nm)	50	50
CSL doping (cm^−3^)	3 × 10^16^	3 × 10^16^
JFET width (μm)	1.3	1.3
P+ region thickness (μm)	1.7	1.7
P+ region doping (cm^−3^)	1 × 10^19^	1 × 10^19^
N base length (μm)	0.35	−
N base thickness (nm)	170	−
N base doping (cm^−3^)	8 × 10^16^	−

**Table 2:** Performance Comparison of the SiC C-MOS and UD-MOS.

Parameters	UD-MOS	C-MOS
*R*_ON_ (mΩ·cm^2^)	35.48	34.99
*V*_ON_ (V)	−1.11	−2.77
*BV* (V)	8317	8217
*V*_TH_ (V)	5.2	5.2
*I*_RRM_ (A/cm^2^)	54	176
*C*_GS_ (nF/cm^2^)	18.1	37.9
*C*_DS_ (pF/cm^2^)	149	149
*C*_GD_ (pF/cm^2^)	4.01	4.43
*Q*_RR_ (μC/cm^2^)	1.22	5.01
*Q*_G_ (nC/cm^2^)	566	970
*Q*_GD_ (nC/cm^2^)	109	121
*R*_ON_ × *Q*_GD_ (mΩ·μC)	3.87	4.24
*E*_ON_ (mJ/cm^2^)	3.8	5.71
*E*_OFF_ (mJ/cm^2^)	3.36	4.38

## Data Availability

Data are contained within the article.
